# Clinical and radiographic outcome of dynamic cervical implant (DCI) arthroplasty for degenerative cervical disc disease: a minimal five-year follow-up

**DOI:** 10.1186/s12891-018-2017-7

**Published:** 2018-04-04

**Authors:** Lin-nan Wang, Bo-wen Hu, Lei Wang, Yue-ming Song, Xi Yang, Li-min Liu, Hao Liu

**Affiliations:** Department of Orthopedics, West China Hospital, Sichuan University, 37 Guoxue Rd, Chengdu, 610041 China

**Keywords:** Dynamic cervical implant, Degenerative cervical disc disease, Heterotopic ossification, Anterior migration, Subsidence

## Abstract

**Background:**

To evaluate the mid- to long-term clinical and radiographic outcomes of anterior cervical discectomy and dynamic cervical implant (DCI) arthroplasty for degenerative cervical disc disease.

**Methods:**

From April 2010 to October 2010, 38 patients with single- or double-level cervical disc herniation underwent anterior cervical discectomy and DCI arthroplasty. The clinical results and radiographic outcomes of these 38 patients (42 levels) were retrospectively evaluated. The clinical results included the visual analogue scale, Japanese Orthopaedic Association score, Neck Disability Index score, 36-item short form health survey questionnaire, and incidences of complications and neurological deterioration. Radiographic results including cervical alignment, intervertebral height, cervical range of motion (ROM), ROM of the functional spinal unit, adjacent intervertebral ROM, migration, subsidence, and heterotopic ossification (HO) were assessed on plain radiography, three-dimensional computed tomography, and magnetic resonance imaging.

**Results:**

The mean follow-up period was 72.3 months (range 68–78 months). During follow-up, all patients showed significant improvements in the visual analogue scale score, Japanese Orthopaedic Association score, Neck Disability Index score, 36-item short form health survey physical component summary score and mental component summary score. The ROM of the functional spinal unit was partly reduced. The DCI migrated forward in 10 of 42 (23.8%) cases, and HO was detected in 24 of the 42 (57.1%) DCI segments. Subsidence was observed in 14 of 42 (33.3%) DCI segments. Two patients experienced symptom recurrence, and were treated conservatively.

**Conclusions:**

The clinical efficacy of DCI arthroplasty was maintained during mid- to long-term follow-up. HO formation is a common phenomenon, leading to a substantial decrease in ROM at the index level and recurrence of neurological symptoms. The incidence of implant subsidence and migration is relatively high, leaving a potential risk of symptoms at the index level and adjacent segment degeneration. We consider that the first choice for patients with degenerative cervical disc disease should still be total disc replacement or anterior cervical discectomy and fusion, rather than DCI arthroplasty.

## Background

The standard anterior surgical procedure for degenerative cervical disc disease is currently anterior cervical discectomy and fusion (ACDF). ACDF achieves a satisfactory clinical outcome and fusion rate; however, it also increases the motion and intradiscal pressure of the adjacent segments [1, 2], resulting in adjacent segment degeneration (ASD) [3, 4]. This complication led to the search for motion-preserving alternative procedures that could provide sufficient stability and simultaneously decrease the rate of ASD [5–7]. The most common non-fusion technique is total disc replacement (TDR). Several randomized controlled trials have reported that TDR has equivalent or superior clinical outcomes and a lower reoperation rate for surgical or adjacent segments than ACDF [8–10]. However, some studies reported that TDR has limitations such as a high rate of heterotopic ossification (HO) and spontaneous fusion [11–13].

The first-generation dynamic cervical implant (DCI) was designed by Matgé in 2002. The second-generation DCI was produced by Paradigm Spine (New York, NY, USA) in 2005, and has been used in clinical practice since 2008 [14]. Several reports have focused on the clinical and radiographic outcomes of DCI cases [15–18]; all such studies achieved satisfactory clinical and radiographic outcomes during an average follow-up period of 24 months. However, mid- to long-term follow-up results are still lacking. Therefore, we retrospectively investigated the clinical and radiographic outcomes of patients who underwent DCI arthroplasty for degenerative cervical disc disease and had a minimum follow-up of 5 years. Clinical and radiographic analyses were used to investigate the mid- to long-term efficacy of the second-generation DCI product.

## Methods

This was a retrospective study. Between April 2010 to October 2010, 38 patients with single- or double-level degenerative disc disease underwent anterior cervical discectomy and DCI arthroplasty in our department. These patients were those aged over 18 years with single- or double-level cervical disc herniation for whom conservative treatment had failed. Exclusion criteria are shown in Table 1. The diagnosis and intended operative level(s) were determined from the combination of medical history, physical examination, and radiographic imaging including preoperative plain radiography (in the lateral, flexion, and extension positions), three-dimensional computed tomography (3D-CT), and magnetic resonance imaging (MRI). The general characteristics of these patients are shown in Table 2.

**Table 1:** Exclusion criteria for anterior cervical discectomy and DCI arthroplasty

Exclusion criteria	
Segmental instability (intervertebral motion>11°or 3 mm)	
Advanced degenerative changes (marked reduction or absence of intervertebral motion or height)	
Facet joint arthrosis or spondylosis	
Cervical kyphosis	
Active infection	
Osteoporosis	
Inflammatory spondloarthropathies such as ankylosing spondylitis or rheumatoid arthritis	
Expected cord edema and myelomalacia	
Known allergy to titanium	
Previous cervical spine surgery

**Table 2:** General information of involved patients

General information	Data
Number of patients	38
Mean age in years (range)	56.8(36~ 78)
Gender (male/female)	18/20
Mean follow-up length in months (range)	72.3(68–78)
Pathogenesis	
Simple disc herniation	18
Combined osteophyte formation	20
Diagnosis	
Radiculopathy	15
Myelopathy	11
Myeloradiculopathy	12
Implanted levels	
C3~ 4	3
C4~ 5	16
C5~ 6	18
C6~ 7	5
In total	42
Operation levels	
1 level	34
2 levels	4

### Surgical procedure

All surgeries were performed by the same spine surgeon, who had over 20 years of experience. Surgeries were conducted using a standard anterior cervical approach and discectomy. Under general anesthesia, the patients were placed in the supine position with the neck slightly extended. Through a right cervical incision, the target segment was exposed with the assistance of intraoperative C-arm X-ray. The anterior longitudinal ligament was opened, and the discectomy procedure was then performed. Cartilaginous endplates and osteophytes on the posterior edge of the vertebral body were excised with a high-speed drill while the bony endplates were protected properly. After sufficient decompression, an appropriately sized DCI was implanted into the intervertebral space under X-ray monitoring. The distance between the anterior/posterior edge of the DCI and the edge of the vertebral body was controlled within a range of 2–3 mm, and the lateral boundary did not exceed the Luschka joint. A drainage tube was left in the wound, and removed within 24 h. Patients were braced in a cervical collar for 1 week, and were then permitted to begin gradual mobilization under the guidance of doctors.

### Evaluation

#### Clinical outcomes

As standard procedure in our department, all patients with degenerative cervical disc disease undergo a neurological examination and complete an extensive questionnaire, including the visual analogue scale (VAS), Japanese Orthopaedic Association (JOA) score, Neck Disability Index (NDI) score, and 36-Item Short Form Health Survey questionnaire (SF-36) preoperatively, at postoperative 1, 12, and 24 months, and at final follow-up.

#### Radiological indications

**Fig. 1:**
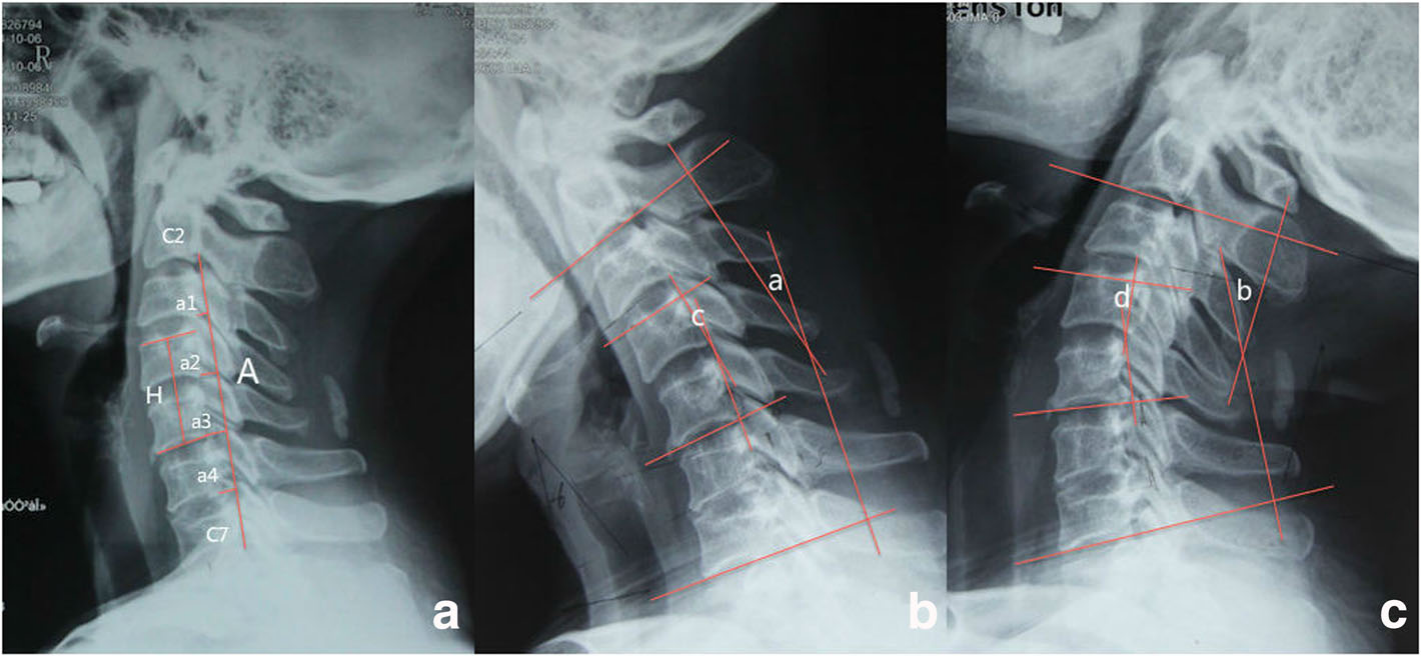
Lateral radiograph showing evaluation of the cervical curvature index (CCI = (a1 + a2 + a3 + a4)/A), intervertebral height (H), the ROM of cervical spine (**a + b**), and the ROM of FSU (**c + d**)

Plain radiography (in the lateral, flexion, and extension positions) and 3D-CT were conducted preoperatively, at 6, 12, and 24 months postoperatively, and at final follow-up. Pre- and postoperative cervical lordosis was measured on lateral radiographs using the cervical curvature index (Fig. 1), as described by Ishihara [19]. The intervertebral height was measured as the distance between the midpoint of the upper endplate of the upper vertebral body and the midpoint of the inferior endplate of the lower vertebral body (Fig. 1). The cervical spine range of motion (ROM), ROM of the functional spinal unit (FSU), and adjacent intervertebral space were calculated using the Cobb’s method on full flexion and extension radiography [20] (Fig. 1). Migration and subsidence of the implant were measured and recorded. Subsidence was defined as a loss of height of more than 3 mm [21]. HO was also recorded.

Data were expressed as the mean ± standard deviation. Results were analyzed statistically with the paired Student’s t-test using SPSS 21.0 software (SPSS Inc., Chicago, Illinois). A *p* value of less than 0.05 was taken to indicate a significant difference.

## Results

The mean follow-up period was 72.3 months (range 68–78 months). In total, 42 levels from 38 patients were included. There were no surgical complications such as wound infection, leakage of cerebrospinal fluid, dysphagia, and/or hoarseness detected throughout the entire follow-up period. During the follow-up period, two patients (5.2%) experienced symptom recurrence at the index level due to a newly-emerging osteophyte at the posterior border of the vertebra; these symptoms were relieved after conservative treatment. A total of six adjacent operation levels had ROM > 11°, indicating that the adjacent level was unstable, but there were no symptoms.

### Clinical outcomes

**Fig. 2:**
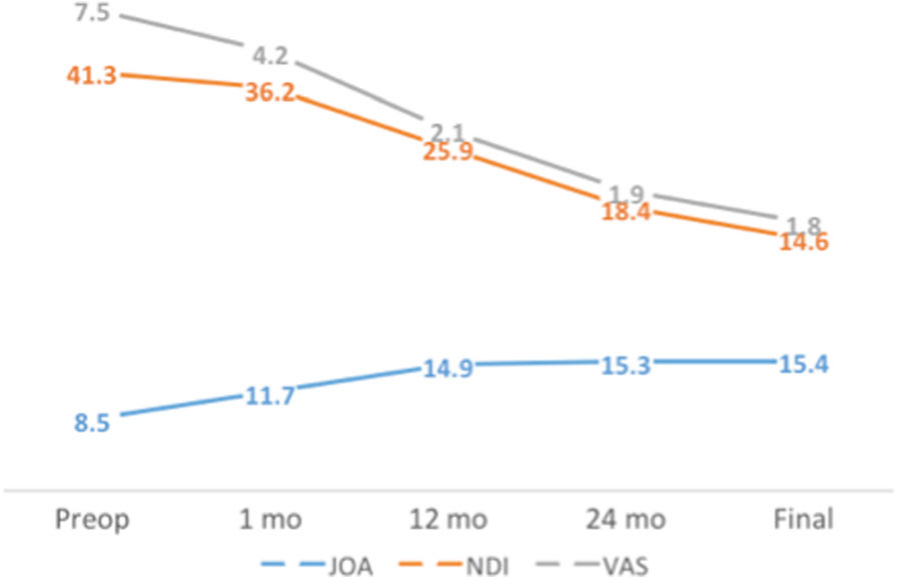
JOA score, NDI score and VAS score of different time-points for involved patients

**Fig. 3:**
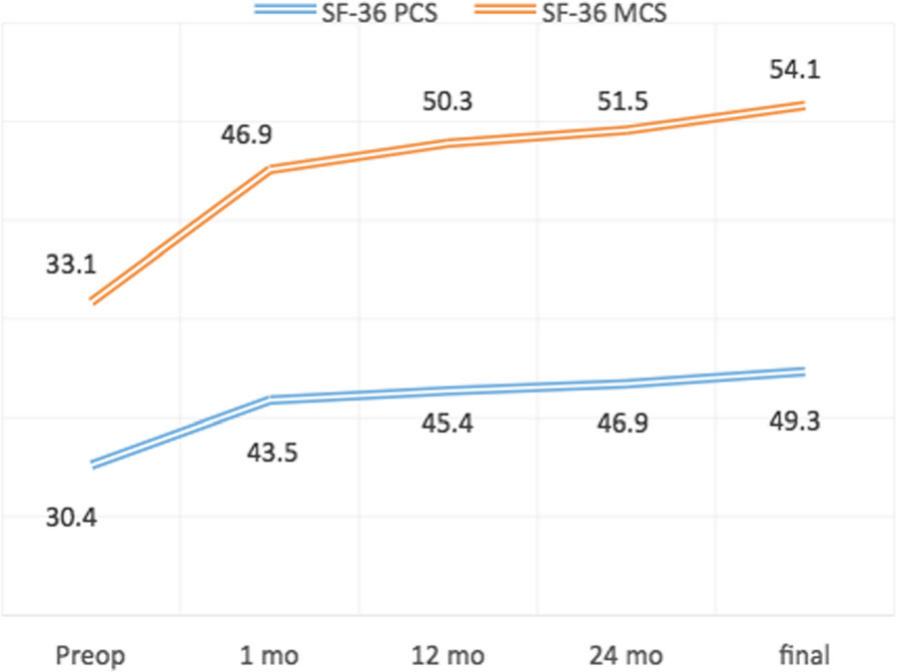
SF-36 PCS and SF-36 MCS of different time-points for involved patients

All patients showed significant postoperative improvement in neurological symptoms. The clinical parameters improved significantly after surgery, and the effect remained at final follow-up. The mean JOA score significantly increased from 8.5 ± 1.4 preoperatively to 15.4 ± 1.8 at final follow-up (Fig. 2). The recovery rate of the JOA score was 81.1%, which satisfied the criterion for clinical efficacy. The mean NDI score significantly decreased from 41.3 ± 4.2 preoperatively to 14.6 ± 3.4 at final follow-up (Fig. 2). The average VAS score significantly decreased from 7.5 ± 0.5 preoperatively to 1.8 ± 0.4 at final follow-up (Fig. 2). The SF-36 physical component summary (PCS) score increased significantly from 30.4 ± 5.2 preoperatively to 49.3 ± 6.7 at final follow-up (Fig. 3). The SF-36 mental component summary (MCS) score increased significantly from 33.1 ± 5.1 preoperatively to 54.1 ± 7.6 at final follow-up (Fig. 3).

### Radiographic analysis

**Fig. 4:**
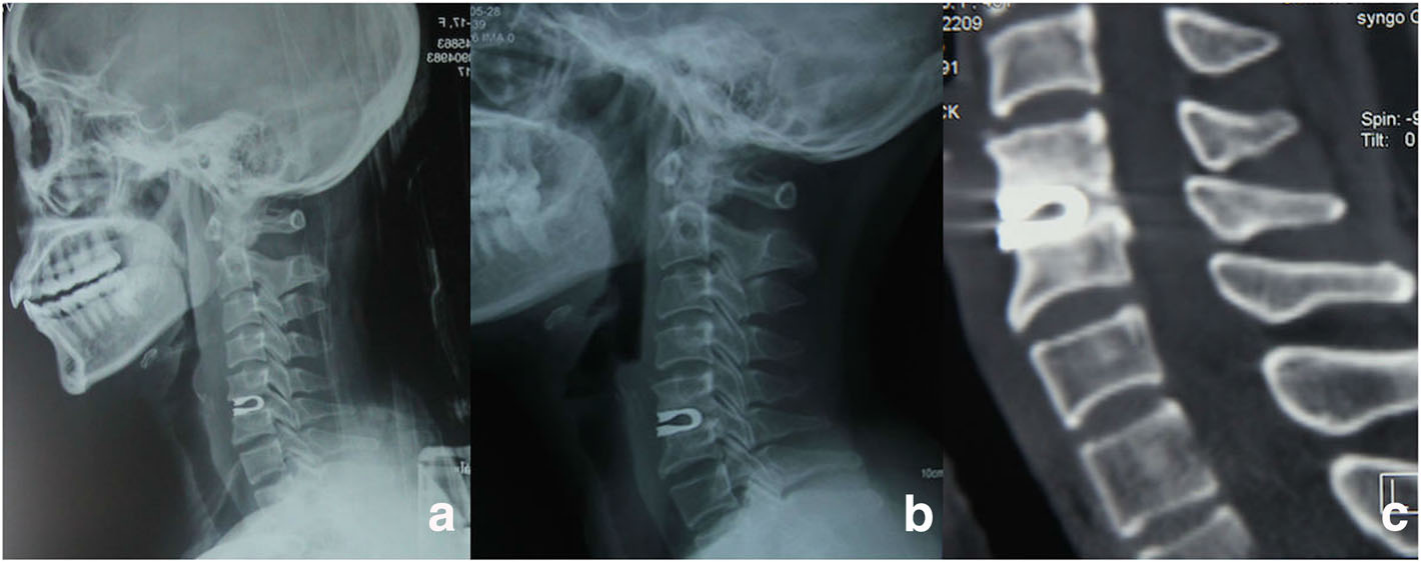
A 39-year old female patient diagnosed C5/6 disc herniation underwent anterior C5/6 discectomy and DCI arthroplasty. Lateral radiographic image of 1-month postoperative (**a**) showed DCI implant was at the appropriate position. However lateral radiographic image and sagittal CT (**b, c**) at the final follow-up illustrated anterior migration of DCI implant with subsidence

**Fig. 5:**
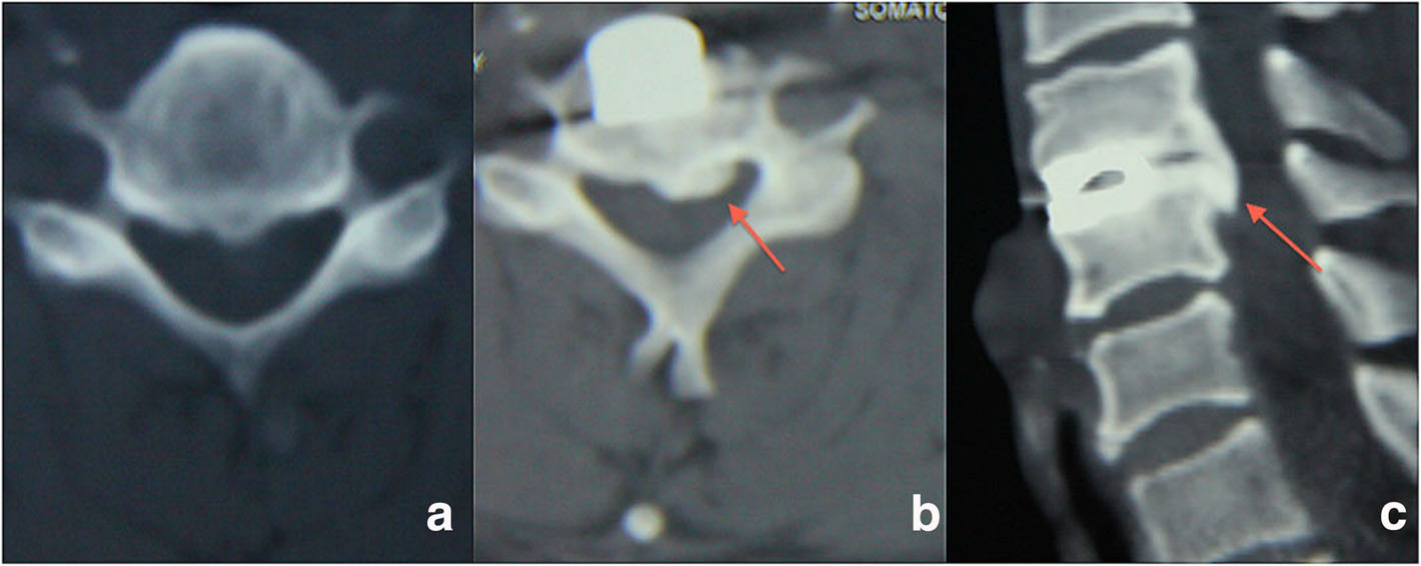
A 41-year old male patient diagnosed C4/5 disc herniation underwent anterior C4/5 discectomy and DCI arthroplasty. Transaxial CT of preoperative (**a**) and final follow-up (**b**) showed HO formation at the index level during follow-up. Sagittal CT at the final follow-up (**c**) illustrated the formation of an ossified bridge, which would restrict ROM of FSU. Also, the osteophyte narrowed the nerve root canal, causing pain and numbness of left arm. Symptom relieved after conservative treatment

**Fig. 6:**
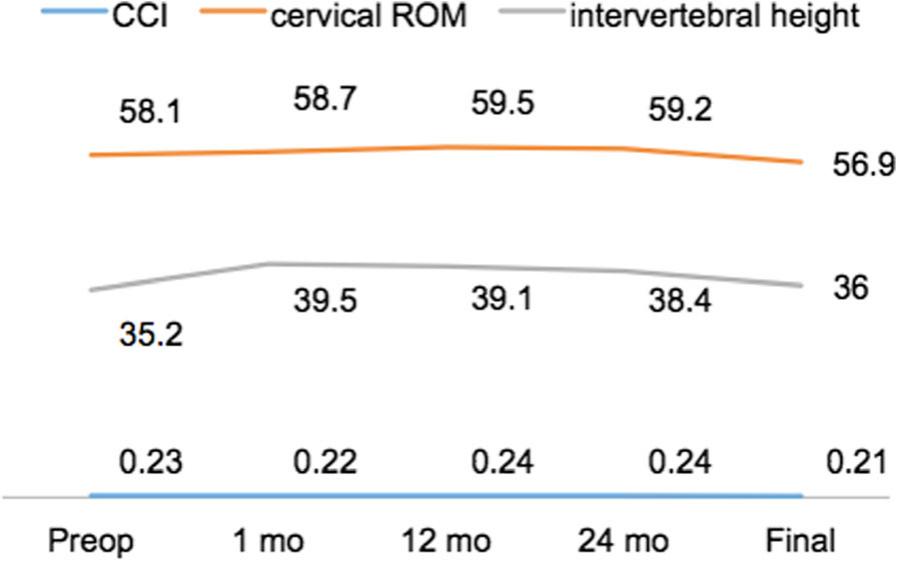
CCI, cervical ROM and intervertebral height of different time-points for involved patients

Two spine surgeons evaluated all radiographs independently. When there was a difference in opinion between the two surgeons, an additional radiologist made the final decision. Ten of the 42 (23.8%) DCIs migrated forward (Fig. 4). HO was detected in 24 of the 42 (57.1%) DCI segments (Fig. 5). The mean cervical curvature index (CCI) was 0.23 ± 0.04 preoperatively, and 0.21 ± 0.04 at final follow-up (Fig. 6); however, this change was not significant.

**Fig. 7:**
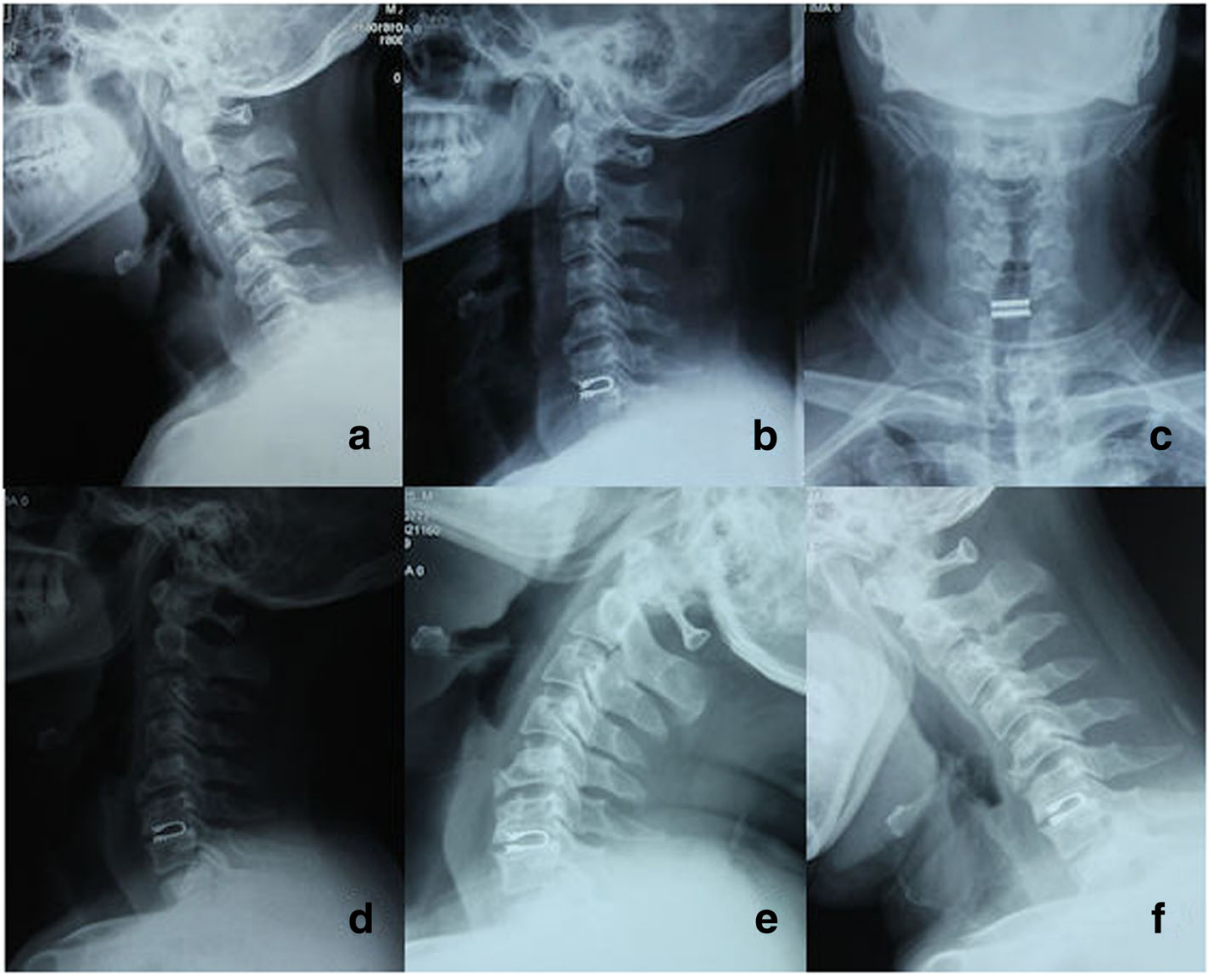
A 36-year old male patient diagnosed C6/7 disc herniation underwent anterior C6/7 discectomy and DCI arthroplasty. Preoperative lateral radiographic image (**a**) showed narrowed intervertebral space. 1 month postoperative X-ray image (**b, c**) demonstrated partial restoration of intervertebral height and appropriate position of DCI implant. However, Lateral and flexion-extension radiographic image at the final follow-up (**d, e, f**) illustrated subsidence of the DCI implant with decrease of the intervertebral height and ROM of FSU

The average intervertebral height on standard lateral radiographs increased from 35.2 ± 3.2 mm preoperatively to 39.5 ± 2.8 mm at 1 month postoperatively and 38.4 ± 3.1 mm at 24 months postoperatively, but decreased to 36.0 ± 3.5 mm at final follow-up (Fig. 6); there was a significant difference between the value at 1 month postoperatively and that at final follow-up (*p* < 0.05). Subsidence occurred in 14 of 42 (33.3%) DCIs. An image from a representative patient is shown in Fig. 7.

**Fig. 8:**
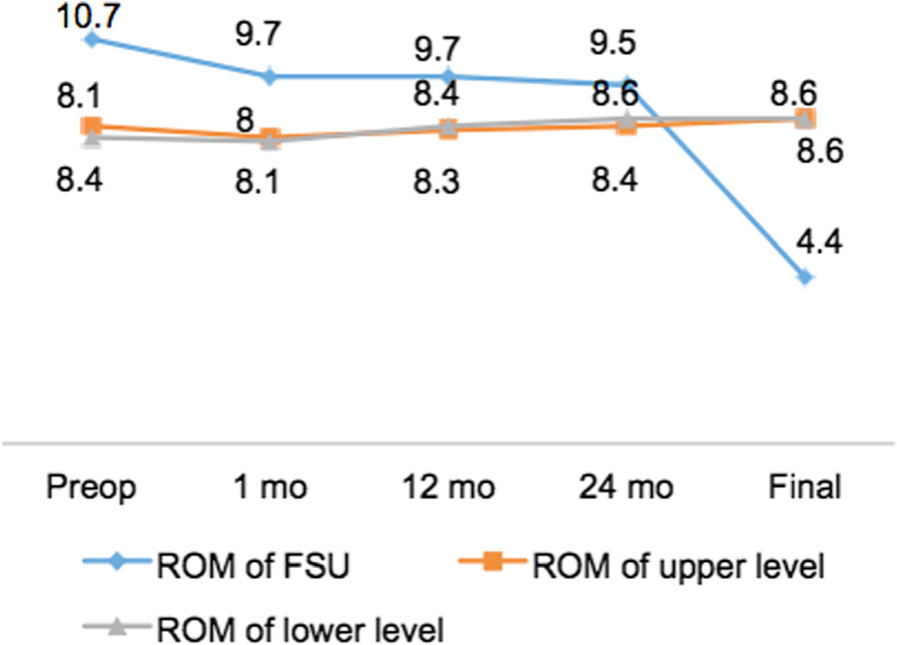
ROM of FSU, upper level and lower level of different time-points for involved patients

The ROM of the cervical spine was 58.1° ± 5.8° preoperatively, 59.2° ± 6.7° at 24 months postoperatively, and 56.9° ± 7.1° at final follow-up (Fig. 6). There was no significant change in ROM during the follow-up period. The average ROM of the FSU was 10.7° ± 2.6° preoperatively, 9.5° ± 2.1° at 24 months postoperatively, and 4.4° ± 1.3° at final follow-up (Fig. 8). The elastic effect was well maintained at the 24-month follow-up, but had significantly decreased at the final follow-up (p < 0.05). The ROM of the upper adjacent operation level was 8.4° ± 2.4°preoperatively, and 8.6° ± 2.5°at final follow-up. The ROM of the lower adjacent operation level was 8.1° ± 1.9°preoperatively, and 8.6° ± 2.5°at final follow-up (Fig. 8). There was no significant change in the ROM of the upper and lower adjacent levels from preoperatively to final follow-up.

## Discussion

DCI arthroplasty is a non-fusion technique that was first introduced by Matgé in 2002, before being improved and introduced into clinical practice by Paradigm Spine (New York, NY, USA) [14]. The two main characteristics of DCI are the U-shaped appearance with runcinate teeth at the anterior edge, and the axial elasticity. Compare with TDR, DCI arthroplasty has several reported advantages: 1) DCI arthroplasty has a wider range of indications and is a relatively simple surgical technique [18], 2) DCI functions as a shock absorber that limits axial rotation and lateral bending, thus exacerbating facet joint stress [17], 3) DCI allows for axial compression in flexion and limited extension, with motion at the index level relatively close to the intact value [22], and 4) there is no grinding of the metal when the DCI functions, and so there is no local or systemic reaction to debris [18].

Several studies have evaluated the clinical efficacy and radiographic outcomes of patients who have undergone DCI arthroplasty. Matgé et al. reported that all 47 patients who underwent DCI arthroplasty achieved satisfactory or somewhat satisfactory clinical outcomes during 2 years of follow-up; three of the 47 patients experienced implant subsidence, and 12 patients had major HO that resulted in less ROM [17]. Li et al. demonstrated that DCI arthroplasty and ACDF had the same effect in improving and maintaining clinical functions, but DCI arthroplasty resulted in a better overall cervical or segmental ROM; no subsidence of DCI was detected during the 2-year follow-up [18]. Liu et al. reported that patients who underwent TDR had a higher incidence of HO than those who underwent DCI arthroplasty during a 2-year follow-up period, but other clinical and radiographic outcomes were similar, and no DCI subsidence was detected [15]. In our previous report on the present series of patients, both clinical and radiographic outcomes were satisfactory, and were maintained for more than 2 years of follow-up [16]. The outcomes of these reports all indicate that DCI is an effective technique for treating degenerative cervical disc disease (mostly single level) in the short-term, but mid- to long-term follow-up data is still lacking.

In the present study, a minimum 5-year follow-up of 38 cases showed that satisfactory clinical outcomes were obtained irrespective of whether the patients had radiculopathy or myelopathy. Compared with preoperatively, there were significant improvements at final follow-up in VAS scores for pain in the upper body, neck, and shoulder, NDI score, JOA score, and SF-36 outcomes; the average ameliorative rate of the JOA score was 81.1%. Although DCI subsidence was detected, clinical efficacy was maintained during follow-up. The present results suggest that neurological improvement depends mainly on thorough decompression.

Motion preservation is the main purpose of the non-fusion technique. DCI arthroplasty is reportedly effective in preserving and maintaining the ROM of the cervical spine or the index level within 2 years of follow-up [15–18]. In the present study, the ROM of the index level was well maintained during the first 2 years of follow-up, but had significantly decreased at final follow-up. We consider that this may have been due to HO formation at the anterior or posterior border of the operation intervertebral space, and/or implant subsidence.

HO is reportedly very common after TDR of the cervical spine, but the mechanism is still uncertain [23]. The underlying cause of HO may be subchondral bleeding [24, 25]. HO formations are commonly seen in studies of DCI arthroplasty [15–17], but the previously reported incidences of HO are not as high as those seen in the present study (57.1%). We suggest that this may be due to the following two reasons: 1) the present study had a longer follow-up duration than these previous studies, and 2) we used a high-speed burr to remove the cartilage endplate intraoperatively, which may injure the bony endplate and cause subchondral bleeding. HO formations restrict segmental ROM, especially when an ossified bridge is formed at the index level. Furthermore, HO at the posterior edge of the vertebra may cause stenosis of the spinal canal or the intervertebral foramen area, leading to recurrence of symptoms. Two of the present patients (5.2%) experienced symptom recurrence at the index level due to newly-emerged osteophytes during follow-up, but these symptoms resolved after conservative treatment.

Severe implant subsidence not only causes a decrease in ROM at the index level, but also leads to a loss of vertebral height of the operated segment; the incidence of subsidence in the present study was 33.3% at final follow-up. Migration was detected in ten of the 42 DCI segments, all of which had migrated forward. Furthermore, migration was only seen at the subsided levels. The incidence of subsidence in the present study is much higher than that reported in studies on TDR [23]. Unlike TDR, the anterior runcinate teeth of the DCI can help maintain stability of the operated segment, which supposedly prevents the implant from migrating or dropping off. However, the elasticity modulus of titanium alloy is much higher than that of the cortical bone of the cervical vertebrae. A relatively high degree of local stress placed on the endplate by the runcinate teeth may cause destruction of the endplate, especially in patients with osteoporosis during follow-up. Moreover, DCI allows flexion-extension motion of the operated segment, which increases the local stress at the DCI–endplate interface. We consider that subsidence occurred mainly because of persistent local stress at the segmental endplates, especially the anterior portion where the DCI–endplate interface was located. In addition, iatrogenic bony endplate injury may sometimes occur during the excision of the cartilaginous endplates. Regarding the DCI migration, the orientation of the runcinate teeth of the DCI is anteroinferior/anterosuperior, which facilitates the anterior migration during subsidence. Theoretically, a decreased intervertebral height causes reduction of the intervertebral foramen area, which is a factor affecting the formation of radiculopathy. However, no patient in the present study has so far experienced numbness, neuralgia, or paralysis of the upper extremities.

Regarding the decrease in local ROM, the index level is likely a fused part, thus leading to the likelihood of a compensatory increase in ROM at the adjacent segments. A total of six adjacent levels in the present study had a ROM > 11°, indicating instability, but these cases were asymptomatic.

The present study had several limitations. These include the lack of a control group, the relatively small sample size, and the fact that all cases were from a single center. Additionally, a longer follow-up duration is still needed to evaluate whether ASD develops over the long-term, and to evaluate the impact of implant subsidence.

## Conclusion

DCI arthroplasty is a non-fusion surgical method for treating cervical spine degeneration. In the present study, clinical efficacy was maintained during mid- to long-term follow-up. HO formation is common at final follow-up, leading to a significant decrease in ROM at the index level, and a potential risk of spinal cord or nerve root compression. The incidence of implant subsidence and migration after DCI arthroplasty is relatively high, carrying a potential risk of ASD and reduction of the intervertebral foramen area. Based on the present results, we suggest that TDR or ACDF should still be the first choice for patients with degenerative cervical disc disease, rather than DCI arthroplasty.

## Abbreviations

ACDF: Anterior cervical discectomy and fusion
ASD: Adjacent segment degeneration
CCI: Cervical curvature index
CT: Computed tomography
DCI: Dynamic cervical implant
FSU: Functional spinal unit
HO: Heterotopic ossification
JOA: Japanese orthopaedic association
MCS: Mental component summary
MRI: Magnetic resonance imaging
NDI: Neck disability index
PCS: Physical component summary
ROM: Range of motion
SF-36: 36-Item short form
TDR: Total disc replacement
VAS: Visual analogue scale
